# Corrected Serum Ionized Calcium as a Risk Factor Related to Adult Dyslipidemia

**DOI:** 10.3389/fcvm.2022.916991

**Published:** 2022-07-06

**Authors:** Ke Yun, Shuang Zhang, Xiaotao Yang, Dongliang Man, Jialiang Yao, Wei Wang, Xiaoxu Han

**Affiliations:** ^1^National Clinical Research Center for Laboratory Medicine, The First Affiliated Hospital of China Medical University, Shenyang, China; ^2^Department of Laboratory Medicine, The First Affiliated Hospital of China Medical University, Shenyang, China; ^3^Units of Medical Laboratory, Chinese Academy of Medical Sciences, Shenyang, China; ^4^NHC Key Laboratory of AIDS Immunology, China Medical University, Shenyang, China; ^5^Department of Physical Examination Center, The First Affiliated Hospital of China Medical University, Shenyang, China

**Keywords:** corrected serum ionized calcium, dyslipidemia, dose-response relationship, stratified analyses, non-linear association

## Abstract

**Background:**

Dyslipidemia is a significant threat to global public health due to its pivotal role as a cardiovascular disease (CVD) risk factor. Calcium is a critical nutritional element required for electrical signal transduction and muscle and heart function, and calcium supplementation is widespread in the general population. However, associations between serum calcium and serum lipid profiles remain conflicting. Considering ionized calcium [Ca(2+)] is the best measure of active serum calcium and the lack of Ca(2+) analyzers, we aimed to examine the independent and joint associations between serum ionized calcium corrected by albumin ([Ca2+]corr) and the known modifiable risk factors and dyslipidemia.

**Methods:**

We collected physical examination records, including demographic, anthropometric, laboratory tests, and clinical characteristics from individuals who had health checkups in 2019 at the health examination center of the First Affiliated Hospital of China Medical University. Subjects were categorized into Q1–Q4 groups using [Ca2+]corr quartiles, and odds ratios (ORs) with 95% confidence intervals (CIs) for dyslipidemia and associated components were calculated using logistic regression. We also performed non-linear and threshold effect analyses of [Ca2+]corr and triglyceride (TG), total cholesterol (TC), high-density lipoprotein cholesterol (HDL-C), low-density lipoprotein cholesterol (LDL-C), and non-high-density lipoprotein cholesterol (Non-HDL-C) levels.

**Findings:**

Of 5,416 individuals aged 18–92 years, multivariable-adjusted models showed that ORs for dyslipidemia increased gradually with elevated [Ca2+]corr levels. Logistic regression analyses demonstrated that [Ca2+]corr levels were associated with the increased odds of dyslipidemia (per 1 mmol/L increase: OR = 3.53, 95% CI: 1.56–8.00, *P* < 0.001). When compared with individuals in the Q1 group, those in groups Q3 and Q4 had significantly higher dyslipidemia odds (OR_*Q*3 vs. Q1_ = 1.20, 95% CI: 1.01–1.42; OR_*Q*4 vs. Q1_ = 1.31, 95% CI: 1.10–1.56, all *P* < 0.05). Furthermore, a linear, positive relationship between [Ca2+]corr levels and dyslipidemia odds was observed (P for non-linear trend = 0.506), and the optimal cut-off point of [Ca2+]corr for dyslipidemia management was 2.26 mmol/L. A modifiable effect of albumin on the relationship between [Ca2+]corr and dyslipidemia odds was also found (P for interaction = 0.014). High [Ca2+]corr levels were positively associated with elevated TC, LDL-C, and Non-HDL-C but inversely associated with decreased HDL-C odds. Moreover, Locally weighted regression (Loess) analyses showed a non-linear, positive relationship between [Ca2+]corr and TG, TC, HDL-C, LDL-C, and Non-HDL-C levels.

**Interpretation:**

Corrected serum ionized calcium was positively associated with increased odds of dyslipidemia and elevated TC, LDL-C, and Non-HDL-C, but inversely associated with the odds of decreased HDL-C.

## Introduction

Dyslipidemia is a significant threat to global public health due to its pivotal role in cardiovascular disease (CVD) (1, 2). The condition is characterized by one or more metabolic disorders involving triglycerides (TG), total cholesterol (TC), high-density lipoprotein cholesterol (HDL-C), and low-density lipoprotein cholesterol (LDL-C) (3). With accelerated urbanization, industrialization, and westernized lifestyles, China rapidly encounters a heavy burden of serum lipid disorders. A national survey in China, including 163,641 adults, showed that the prevalence of high TC, high LDL-C, low HDL-C, and high TG was 6.9, 8.1, 20.4, and 13.8% in China (4). Thus, identifying and quantifying associated risk factors with serum lipids is warranted for the targeted prevention of dyslipidemia and CVD events.

Calcium is a crucial nutritional element required for electrical signal transduction (5) and muscle and heart function, and calcium supplementation is widespread in the general population (6). Some studies reported that appropriate calcium levels were beneficial for fat metabolism (7). However, some other studies suggested calcium may be associated with an increased risk of CVD events (8, 9), such as coronary artery calcification (10). Therefore, the literature on the relationship between serum calcium and serum lipids is inconclusive, and warrants more comprehensive analyses.

Considering ionized calcium (Ca2+) is the best measure of active serum calcium, the formula of albumin corrected serum calcium was used to estimate ionized calcium levels (11). This study investigated whether corrected serum ionized calcium ([Ca2+]corr) was associated with higher odds of dyslipidemia (and associated components), including elevated TG, elevated TC, reduced HDL-C, elevated LDL-C, and elevated Non-HDL-C levels. We sought to ascertain the optimal cut-off value of corrected serum ionized calcium for dyslipidemia odds and investigate the dose-response relationship between corrected serum ionized calcium and serum lipid parameters in Chinese adults.

## Materials and Methods

### Study Population

We retrospectively collected datasets containing 44,061 subjects’ health check-up medical records in 2019 from the health examination center of the First Affiliated Hospital of China Medical University (Shenyang, China). The database mainly includes medical examination records of employees from different organizations, subjects who are going to work, students who are going to school, and an individual who voluntarily attends a health check-up, etc. The medical examination center offers different medical examination packages, and the physical examination items to be carried out are independently selected by the employees or individual themselves. The health check-up database lacked information on medication use and other diseases that might affect laboratory testing, although few people take medication in the last 3 days of physical examination. The inclusion criteria of the participants included: (1) aged 18 years or older and (2) received serum calcium and serum lipid testing. The exclusion criteria included: (1) participants who lacked serum calcium or serum lipid measurements, (2) individuals aged under 18 years.

### Measurements

Selected individuals were assessed for demographics, anthropometrics, laboratory and clinical parameters during their health check-ups. Firstly, a health examination form was used to collect demographics on age and sex. Secondly, height, weight and waist circumstance were measured by trained medical staff; participants wore light clothing and were barefoot. Body mass index (BMI) was then calculated using weight divided by the height squared. Systolic blood pressure (SBP) and diastolic blood pressure (DBP) were measured using an electronic sphygmomanometer while the participant was calmly seated. Thirdly, the subjects were reminded to prepare themselves before the physical examination, including (1) 3 days before the physical examination to pay attention to diet, do not eat too much greasy, indigestible food, and do not drink (2) no water or food on the day of physical examination. Blood samples were taken after an overnight fast in the morning by trained clinical nurses. Samples were centrifuged to separate serum and stored at −80^°^C until required tests. Collected serum biochemical parameters included serum calcium, fasting serum glucose (FSG), TC, TG, HDL-C, LDL-C, alanine aminotransferase (ALT), aspartate aminotransferase (AST), serum creatinine, serum uric acid, and albumin. Routine blood testing included counts for leucocytes, granulocytes, lymphocytes, monocytes, eosinophils, basophils, and red blood cells (RBC). We also tested hemoglobin concentration (HGB), red blood cell specific volume (HCT), mean corpuscular volume (MCV), mean corpuscular hemoglobin (MCH), mean corpuscular hemoglobin concentration (MCHC), platelet counts (PLT), platelet distribution width (PDW), large platelet ratio (P-LCR), plateletcrit (PCT), and mean platelet volume (MPV). The laboratory indicators were tested using the Roche automatic analyzer (Roche Diagnostics, Mannheim, Germany) for biochemistry parameters and Sysmex hematology analyzers (Sysmex Corporation, Kobe, Japan) for hematologic parameters.

### Clinical Definitions

Dyslipidemia was defined by high TC ≥ 5.18 mmol/L (200 mg/dL), and/or high TG ≥ 1.76 mmol/L (150 mg/dL), and/or high LDL-C ≥ 3.37 mmol/L (130 mg/dL), and/or low HDL-C ≤ 1.04 mmol/L (40 mg/dL) according to the guidelines on prevention and management of dyslipidemia in Chinese adults (12). Non-high-density lipoprotein cholesterol (Non-HDL-C) was calculated as TC minus HDL-C. High Non-HDL-C was defined as ≥ 4.1 mmol/L (158 mg/dL) (13). Diabetes was defined as a FSG concentration greater than or equal to 7.0 mmol/L (126 mg/dL), or self-reported diabetes history (14). Hypertension was defined by an SBP ≥ 140 mmHg and/or DBP ≥ 90 mmHg according to Chinese hypertension guidelines (15). The estimated glomerular filtration rate (eGFR) was calculated to reflect the overall index of kidney function using the epidemiology collaboration (EPI)-Asia equation (16, 17).

For male: SCr ≤ 0.9 mg/dL, eGFR = 141 × (SCr/0.9)^–0^.^411^ × (0.993)^Age^ × 1.057;

For male: SCr > 0.9 mg/dL, eGFR = 141 × (SCr/0.9)^–1^.^209^ × (0.993)^Age^ × 1.057;

For female: SCr ≤ 0.7 mg/dL, eGFR = 144 × (SCr/0.7)^–0^.^329^ × (0.993)^Age^ × 1.049;

For female: SCr > 0.7 mg/dL, eGFR = 144 × (SCr/0.7)^–1^.^209^ × (0.993)^Age^ × 1.049;

The units for each index were: age (years), SCr (serum creatinine) (mg/dL), and eGFR (ml/min/1.73 m^2^).

Given that serum calcium changes with albumin concentrations (18), serum calcium was adjusted according to the following formula into corrected serum ionized calcium ([Ca2+]corr); [Ca2+]corr (mmol/L) = serum calcium (mmol/L) + 0.02 × [40 − albumin (g/L)] (11).

### Statistical Analyses

Each subject was assigned a digital code during the study to identify individuals. The columes of variables with missing values > 20% were eliminated. Because physical examinees will choose different physical examination packages, and not all test items will be detected for an individual undergoing medical examination. And then, missing data were excluded by using the listwise method, and outliers of serum calcium were excluded by the Tukey method. Records with complete information on other covariates were also kept for final analyses. The Kolmogorov-Smirnov test was used for normal distribution analyses. Descriptive categorical variables were expressed as numbers and percentages, and continuous variables were described as the median and interquartile range (IQR) or the mean ± standard deviation (SD). Group differences were compared using the Chi-squared test, *t*-test, Mann–Whitney U test or Kruskal–Wallis H test. The stepwise variable selection method was used to select variables in multivariate-adjusted models to avoid over- or under-fitting. The multicollinearity predictor was ruled out with a variance inflation factor ≥ 5. [Ca2+]corr values were taken as continuous/categorical variables to explore associations with dyslipidemia and associated components. [Ca2+]corr levels were divided into four groups according to quartile distributions: Q1 group (1.97–2.20 mmol/L), Q2 (2.20–2.26 mmol/L), Q3 (2.26–2.31 mmol/L), and Q4 (2.31–2.52 mmol/L). Restricted cubic spline regression analyses with four knots were used to assess the dose-response association between [Ca2+]corr values and dyslipidemia. The optimal risk threshold was then determined. In addition, non-linear relationships between [Ca2+]corr and serum lipid component levels were explored using locally weighted regression (Loess) to help interpret findings. Interactions between age, gender, waist circumstance, BMI, eGFR, ALT, AST, albumin and [Ca2+]corr concerning dyslipidemia were also examined. A two-sided *P* < 0.05 value was considered statistically significant. All statistical analyses were performed using R software (version 3.4.0^1^).

### Ethics and Privacy Protection

The ethics committee of the First Affiliated Hospital of China Medical University approved this study (approval No. 2020-323). Informed consent was waived as this was a retrospective study. Individual information was hidden, and privacy was maintained to protect participant information.

## Results

### Demographic and Clinical Characteristics

**FIGURE 1:**
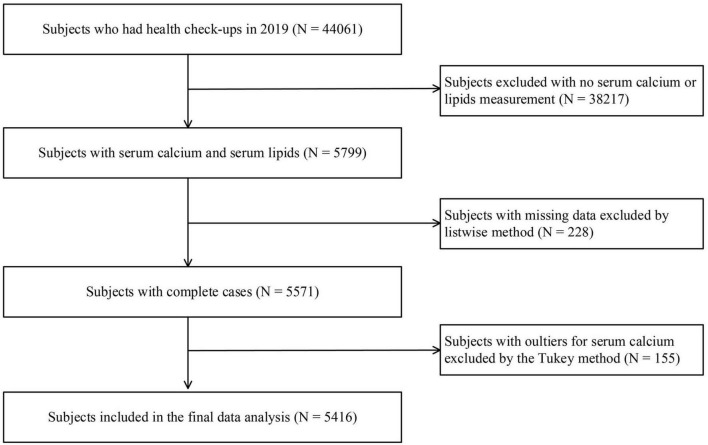
Flow chart of the subject selection process.

A total of 5,416 participants with 42 variables (demographic, anthropometric, laboratory tests, and clinical variables) were included in this study. A flowchart of the selection process is shown in Figure 1. Of these participants aged 18–92 years (2,920 men and 2,496 women), 3,316 (61%) had dyslipidemia. The median serum calcium level was 2.34 mmol/L (IQR: 2.28–2.40 mmol/L), and the median [Ca2+]corr was 2.26 (IQR: 2.20–2.31). When compared with subjects in the Q1 group, individuals in the Q2, Q3, and Q4 group were older. They showed a higher waist circumstance and higher BMI, SBP, DBP, TG, TC, HDL-C, LDL-C, Non-HDL-C, creatinine, FSG, uric acid, AST, ALT, leucocyte, granulocyte, lymphocyte, monocyte, eosinophil, basophil, RBC, HGB, HCT, MCV, MCHC, PLT, PCT, and MPV levels, and lower albumin, PDW and P-LCR levels. These participants also had a high proportion of hypertension, dyslipidemia, high TG, high TC, high LDL-C, and high Non-HDL-C, but a low proportion of reduced HDL-C (all *P* < 0.05) (Table 1).

**TABLE 1:** Participant demographics and laboratory parameters according to [Ca2+]corr quartiles (*N* = 5416).

Variables	Overall	[Ca2+]corr, mmol/L				P for trend
		Q1 (1.97–2.20)	Q2 (2.20–2.26)	Q3 (2.26–2.31)	Q4 (2.31–2.52)	
**Demographic and anthropometric characteristics**						
Participants, *n*		1358	1372	1361	1325	
Male, No. (%)	2,920 (54%)	703 (52%)	767 (56%)	743 (55%)	707 (53%)	0.563
Age, years	51 (41–58)	48 (38–56)	50 (40–58)	52 (44–59)	53 (46–59)	< 0.001
Waist circumstance, cm	83 (76–90)	82 (74–89)	83 (76–90)	84 (77–90)	84 (76–90)	< 0.001
BMI, kg/m^2^	25.0 (22.8–27.3)	24.8 (22.5–27.1)	25.1 (22.7–27.3)	25.1 (22.8–27.5)	25.1 (23.0–27.3)	0.007
SBP, mmHg	127 (115–141)	123 (112–137)	127 (115–140)	129 (117–143)	128 (117–142)	< 0.001
DBP, mmHg	76 (68–85)	74 (66–83)	76 (68–85)	77 (68–86)	77 (69–85)	< 0.001
**Biochemistry testing**						
ALT, U/L	19 (14–29)	18 (13–26)	20 (14–28)	20 (14–30)	21 (15–30)	< 0.001
AST, U/L	19 (16–24)	19 (16–22)	19 (16–23)	20 (17–24)	20 (17–24)	< 0.001
TG, mmol/L	1.27 (0.84–1.93)	1.14 (0.75–1.81)	1.25 (0.83–1.86)	1.32 (0.88–2.04)	1.38 (0.93–2.03)	< 0.001
TC, mmol/L	4.90 (4.32–5.52)	4.68 (4.15–5.25)	4.81 (4.30–5.41)	4.97 (4.43–5.61)	5.17 (4.52–5.74)	< 0.001
HDL-C, mmol/L	1.28 (1.06–1.55)	1.25 (1.04–1.54)	1.29 (1.06–1.54)	1.27 (1.07–1.55)	1.31 (1.08–1.59)	0.001
LDL-C, mmol/L	3.07 (2.57–3.62)	2.90 (2.43–3.45)	3.02 (2.52–3.55)	3.14 (2.66–3.69)	3.25 (2.71–3.80)	< 0.001
Non-HDL-C, mmol/L	3.57 (2.97, 4.21)	3.36 (2.80, 3.97)	3.50 (2.92, 4.12)	3.62 (3.08, 4.26)	3.81 (3.13, 4.42)	< 0.001
Creatinine, μmol/L	63 (52–73)	62 (52–73)	64 (52–74)	63 (52–73)	63 (53–73)	0.161
eGFR, ml/min/1.73 m^2^	113 (103–127)	112 (102–126)	114 (103–128)	114 (103–128)	114 (104–128)	0.201
FSG, mmol/L	5.17 (4.83–5.62)	5.11 (4.79–5.51)	5.14 (4.82–5.60)	5.20 (4.86–5.68)	5.22 (4.88–5.69)	< 0.001
Albumin, g/L	44.00 (42.50–45.60)	44.10 (42.70–45.60)	44.10 (42.40–45.70)	44.00 (42.40–45.60)	44.00 (42.40–45.60)	0.009
Uric acid, μmol/L	323 (265–388)	317 (259–375)	319 (261–388)	323 (267–394)	335 (277–399)	< 0.001
**Routine blood testing**						
Leucocyte, 10^9^/L	5.73 (4.91–6.72)	5.60 (4.80–6.56)	5.74 (4.96–6.69)	5.77 (4.93–6.78)	5.81 (4.97–6.90)	< 0.001
Granulocyte, 10^9^/L	3.15 (2.56–3.88)	3.10 (2.55–3.81)	3.18 (2.59–3.86)	3.19 (2.56–3.93)	3.14 (2.54–3.95)	0.002
Lymphocyte, 10^9^/L	1.96 (1.64–2.35)	1.90 (1.59–2.28)	1.97 (1.64–2.34)	1.96 (1.63–2.35)	2.02 (1.68–2.43)	< 0.001
Monocyte, 10^9^/L	0.39 (0.32–0.48)	0.38 (0.31–0.47)	0.39 (0.32–0.48)	0.39 (0.31–0.48)	0.41 (0.33–0.50)	< 0.001
Eosinophils count, 10^9^/L	0.10 (0.06–0.17)	0.10 (0.06–0.16)	0.10 (0.06–0.17)	0.10 (0.06–0.17)	0.11 (0.07–0.18)	0.003
Basophil count, 10^9^/L	0.020 (0.010–0.030)	0.020 (0.010–0.030)	0.020 (0.010–0.030)	0.020 (0.010–0.030)	0.020 (0.010–0.030)	0.023
RBC, 10^12^/L	4.84 (4.51–5.18)	4.80 (4.45–5.18)	4.84 (4.52–5.16)	4.83 (4.50–5.19)	4.88 (4.57–5.20)	< 0.001
HGB, g/L	146 (134–157)	144 (131–156)	146 (134–156)	146 (135–157)	147 (137–159)	< 0.001
HCT	0.43 (0.41–0.46)	0.43 (0.40–0.46)	0.43 (0.41–0.46)	0.43 (0.41–0.46)	0.44 (0.41–0.47)	< 0.001
MCV, fL	89.5 (86.9–92.0)	88.8 (86.2–91.5)	89.5 (86.8–92.0)	89.6 (87.2–92.2)	90.0 (87.6–92.4)	< 0.001
MCH, pg	336 (328–343)	336 (329–343)	335 (328–343)	336 (329–342)	335 (328–342)	0.166
MCHC, g/L	30.10 (29.10–31.10)	29.90 (28.90–30.90)	30.00 (29.10–31.02)	30.10 (29.20–31.10)	30.20 (29.20–31.10)	< 0.001
PLT, 10^9^/L	241 (206–279)	234 (201–271)	240 (206–279)	243 (210–280)	245 (210–286)	< 0.001
PDW	12.80 (11.70–14.10)	12.90 (11.80–14.10)	12.80 (11.60–14.10)	12.80 (11.60–14.00)	12.70 (11.60–14.00)	0.048
P-LCR	31 (26–36)	31 (27–36)	31 (26–36)	31 (26–36)	31 (26–36)	0.045
PCT	0.26 (0.22–0.30)	0.25 (0.22–0.29)	0.26 (0.22–0.30)	0.26 (0.23–0.30)	0.26 (0.23–0.30)	< 0.001
MPV, fL	10.70 (10.20–11.30)	10.80 (10.20–11.40)	10.70 (10.10–11.30)	10.70 (10.10–11.30)	10.70 (10.10–11.30)	0.032
**Clinical characteristics**						
Hypertension, %	1,587 (29%)	326 (24%)	388 (28%)	453 (33%)	420 (32%)	< 0.001
Diabetes, %	313 (5.8%)	63 (4.6%)	72 (5.2%)	85 (6.2%)	93 (7.0%)	0.004
Dyslipidemia, %	3,316 (61%)	729 (54%)	821 (60%)	872 (64%)	894 (67%)	< 0.001
High TG, %	1,638 (30%)	354 (26%)	381 (28%)	456 (34%)	447 (34%)	< 0.001
High TC, %	2,042 (38%)	378 (28%)	457 (33%)	550 (40%)	657 (50%)	< 0.001
Low HDL-C, %	1,171 (22%)	315 (23%)	312 (23%)	302 (22%)	242 (18%)	0.002
High LDL-C, %	1,919 (35%)	378 (28%)	448 (33%)	498 (37%)	595 (45%)	< 0.001
High Non-HDL-C, %	1,562 (29%)	280 (21%)	351 (26%)	421 (31%)	510 (38%)	< 0.001

*Categorical variables are expressed as n (%); continuous variables are expressed as the median (IQR) or the mean ± standard deviation (SD). [Ca2+]corr, corrected serum ionized calcium; BMI, body mass index; ALT, alanine aminotransferase; AST, aspartate aminotransferase; SBP, systolic blood pressure; DBP, diastolic blood pressure; TC, total cholesterol; TG, triglyceride; HDL-C, high-density lipoprotein cholesterol; LDL-C, low-density lipoprotein cholesterol; Non-HDL-C, non-high-density lipoprotein cholesterol; eGFR, estimated glomerular filtration rate; FSG, fasting serum glucose; RBC, red blood cells; HGB, hemoglobin content; HCT, Hematocrit; MCV, average red blood cell volume; MCH, average red blood cell hemoglobin content; MCHC, average red blood cell hemoglobin concentration; PLT, platelet counts; PDW, platelet distribution width; P-LCR, large platelet ratio; PCT, plateletcrit; MPV, mean platelet volume.*

### Associations Between [Ca2+]corr and Dyslipidemia Odds

**FIGURE 2:**
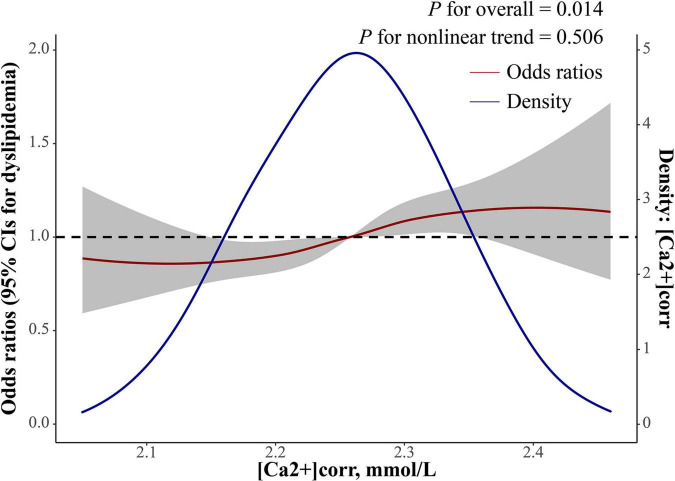
Dose-response relationship between [Ca2+]corr and dyslipidemia odds. The red line with a 95% confidence band is multivariate-adjusted odds ratios using a restricted cubic spline regression with four knots with a polynomial smoother. The blue line is a kernel density plot of [Ca2+]corr. [Ca2+]corr, corrected serum ionized calcium.

As shown (Table 2), logistic regression analyses demonstrated that each 1 mmol/L increment of [Ca2+]corr was associated with an increased odds of dyslipidemia [adjusted odds ratio (AOR) = 3.53, 95% CI: 1.56–8.00, *P* = 0.002] in the multivariate-adjusted model 2. Individuals in Q2, Q3, and Q4 groups had higher dyslipidemia odds in the crude model (OR_*Q*2 vs. Q1_ = 1.29, 95% CI: 1.10–1.50; OR_*Q*3 vs. Q1_ = 1.54, 95% CI: 1.32–1.79; OR_*Q*4 vs. Q1_ = 1.79, 95% CI: 1.53–2.09). After adjusting for gender, age, waist circumference, and BMI in model 1, individuals in Q3 and Q4 had higher dyslipidemia odds when compared with individuals in the Q1 group (OR_*Q*3 vs. Q1_ = 1.32, 95% CI: 1.12–1.55, OR_*Q*4 vs. Q1_ = 1.56, 95% CI: 1.32–1.84). After adjusting for all covariables in model 1 plus clinical and laboratory parameter confounders, including SBP, DBP, a diabetes diagnosis, lymphocytes, basophils, RBC, MPV, FSG, albumin, uric acid, and ALT, participants in Q3 and Q4 groups had a higher dyslipidemia odds in model 2 (OR_*Q*3 vs. Q1_ = 1.20, 95% CI: 1.01–1.42, OR_*Q*4 vs. Q1_ = 1.31, 95% CI: 1.10–1.56). Multivariate-adjusted restricted cubic spline logistic regression analyses with four knots showed a positive linear relationship between [Ca2+]corr and dyslipidemia odds (P for non-linearity = 0.506), and the optimal cut-off value for [Ca2+]corr for dyslipidemia odds equal to 1 was 2.26 mmol/L (Figure 2).

**TABLE 2:** Associations between [Ca2+]corr and dyslipidemia odds in 5,416 adults assessed using logistic regression models.

[Ca2+]corr, mmol/L	*N*	Dyslipidemia	Crude model			Model 1			Model 2	
			OR (95% CIs)	*P*-value	P for trend	OR (95% CIs)	*P*-value	P for trend	OR (95% CIs)	*P*-value	P for trend
per 1 mmol/L increase	5416	3316	17.6 (8.56–36.5)	<0.001	<0.001	8.36 (3.84–18.2)	<0.001	<0.001	3.53 (1.56–8.00)	0.002	0.002
quartiles											
Q1 (1.97–2.20)	1358	729	–			–			–		
Q2 (2.20–2.26)	1372	821	1.29 (1.10–1.50)	0.001		1.16 (0.99–1.37)	0.071		1.13 (0.95–1.34)	0.2	
Q3 (2.26–2.31)	1361	872	1.54 (1.32–1.79)	<0.001		1.32 (1.12–1.55)	0.001		1.20 (1.01–1.42)	0.038	
Q4 (2.31–2.52)	1325	894	1.79 (1.53–2.09)	<0.001		1.56 (1.32–1.84)	<0.001		1.31 (1.10–1.56)	0.003	

*Crude model was unadjusted univariate model.*

*Model 1 was adjusted for gender, age, waist circumference, and BMI.*

*Model 2 was for all covariables in model 1 plus clinical and laboratory parameter confounders, including SBP, DBP, diagnosed as diabetes, lymphocytes, basophils, RBC, MPV, FSG, albumin, uric acid, and ALT. [Ca2+]corr, corrected serum ionized calcium; BMI, body mass index; SBP, systolic blood pressure; DBP, diastolic blood pressure; FSG, fasting serum glucose; RBC, red blood cell count; MPV, mean platelet volume; ALT, alanine aminotransferase; OR, odds ratio; CI, confidence interval.*

### Stratified Analysis Between [Ca2+]corr and Dyslipidemia by Participant Characteristics

We also investigated if the association between [Ca2+]corr and dyslipidemia odds was modifiable by age, gender, waist circumstance, BMI, eGFR, ALT, AST or albumin using stratified analyses. The risk of dyslipidemia was significantly higher among people whose albumin level was higher than 46.6 g/L (P for interaction = 0.014). In subgroup analyses, there were no differences in association between the risk of dyslipidemia and [Ca2+]corr regarding age (*P* = 0.386), gender (*P* = 0.521), waist circumstance (*P* = 0.273), BMI (*P* = 0.891), eGFR (*P* = 0.11), ALT (*P* = 0.591), and AST (*P* = 0.832, Table 3).

**TABLE 3:** Stratified analyses between [Ca2+]corr and dyslipidemia by participant characteristics.

Stratification covariates	No. of participants	[Ca2+]corr, mmol/L				P for Trend	P for Interaction
		Q1 (1.97–2.20)	Q2 (2.20–2.26)	Q3 (2.26–2.31)	Q4 (2.31–2.52)		
**Age, years**							
< 55	3487	1 (Reference)	1.00 (0.82–1.23)	1.16 (0.94–1.44)	1.33 (1.07–1.66)	0.005	0.386
55–64	1354	1 (Reference)	1.59 (1.11–2.30)	1.42 (0.99–2.03)	1.58 (1.11–2.25)	0.044	
≥ 65	575	1 (Reference)	1.32 (0.74–2.37)	1.31 (0.75–2.29)	1.15 (0.65–2.05)	0.8	
**Gender**							
Male	2920	1 (Reference)	1.28 (1.01–1.62)	1.25 (0.98–1.59)	1.47 (1.15–1.88)	0.005	0.521
Female	2496	1 (Reference)	0.98 (0.77–1.26)	1.07 (0.83–1.38)	1.09 (0.85–1.41)	0.4	
**Waist circumstance, cm**							
< 76.8	1806	1 (Reference)	0.95 (0.71–1.26)	1.23 (0.92–1.64)	1.16 (0.87–1.55)	0.14	0.273
76.8–131	1805	1 (Reference)	1.36 (1.02–1.80)	1.34 (1.00–1.78)	1.43 (1.07–1.93)	0.024	
≥ 131.1	1805	1 (Reference)	1.10 (0.79–1.53)	0.97 (0.70–1.35)	1.23 (0.87–1.74)	0.4	
**BMI, kg/m^2^**							
< 25.0	2705	1 (Reference)	1.04 (0.83–1.32)	1.16 (0.91–1.46)	1.24 (0.98–1.58)	0.052	0.891
25–29.9	2227	1 (Reference)	1.18 (0.90–1.55)	1.26 (0.96–1.66)	1.30 (0.98–1.73)	0.057	
≥ 30	484	1 (Reference)	1.19 (0.61–2.31)	0.98 (0.50–1.92)	1.54 (0.73–3.29)	0.4	
**eGFR, ml/min/1.73 m^2^**							
< 60	4318	1 (Reference)	1.03 (0.85–1.25)	1.06 (0.87–1.28)	1.17 (0.96–1.43)	0.12	0.11
≥ 60	1098	1 (Reference)	1.62 (1.08–2.44)	1.61 (1.09–2.39)	1.56 (1.04–2.34)	0.063	
**ALT, U/L**							
< 40	4741	1 (Reference)	1.14 (0.95–1.36)	1.20 (1.00–1.44)	1.29 (1.08–1.55)	0.005	0.591
≥ 40	675	1 (Reference)	1.22 (0.68–2.19)	1.47 (0.82–2.66)	2.00 (1.08–3.77)	0.023	
**AST, U/L**							
< 40	5231	1 (Reference)	1.12 (0.95–1.33)	1.18 (0.99–1.41)	1.30 (1.09–1.55)	0.004	0.832
≥ 40	185	1 (Reference)	1.22 (0.36–4.14)	1.78 (0.50–6.60)	1.26 (0.38–4.12)	0.6	
**Albumin, g/L**							
< 40	1806	1 (Reference)	1.43 (1.07, 1.91)	1.21 (0.91, 1.62)	1.32 (0.98, 1.77)	0.2	0.014
40–46.6	1805	1 (Reference)	0.77 (0.57, 1.04)	0.92 (0.68, 1.24)	1.07 (0.79, 1.46)	0.5	
≥ 46.6	1805	1 (Reference)	1.36 (1.01, 1.82)	1.80 (1.33, 2.46)	1.88 (1.37, 2.57)	< 0.001	

*Adjusted covariates for gender, age, waist circumference, and BMI, SBP, DBP, diagnosed as diabetes, lymphocytes, basophils, RBC, MPV, FSG, fasting serum glucose, albumin, uric acid, and ALT. [Ca2+]corr, corrected serum ionized calcium; BMI, body mass index; SBP, systolic blood pressure; DBP, diastolic blood pressure; RBC, red blood cell count; MPV, mean platelet volume; ALT, alanine aminotransferase; OR, odds ratio; CI, confidence interval.*

### Associations Between [Ca2+]corr and Total Cholesterol, Triglycerides, High-Density Lipoprotein Cholesterol, Low-Density Lipoprotein Cholesterol, and Non-high-Density Lipoprotein Cholesterol Disorders

**FIGURE 3:**
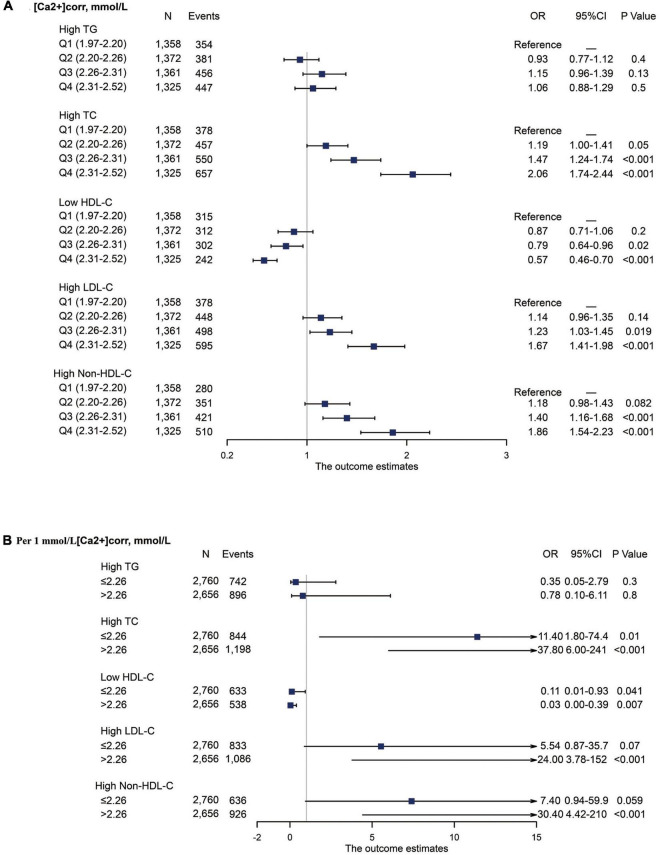
Forest plot of serum lipid disorder odds associated with [Ca2+]corr categories. All analyses were multivariable adjusted. **(A)** Odds associated with four [Ca2+]corr categories. **(B)** Odds associated with a 1 mmol/L [Ca2+]corr increase, stratified by an [Ca2+]corr cut-off value of 2.26 mmol/L. [Ca2+]corr, corrected serum ionized calcium; TC, total cholesterol; TG, triglyceride; HDL-C, high-density lipoprotein cholesterol; LDL-C, low-density lipoprotein cholesterol; Non-HDL-C, non-high-density lipoprotein cholesterol; OR, odds ratio; CI, confidence interval.

Associations between [Ca2+]corr and serum lipid parameters disorders, including high TC, high TG, low HDL-C, high LDL-C, and high Non-HDL-C are shown in Figure 3. As indicated (Figure 3A), multivariable-adjusted ORs of individuals in the Q3 group were 1.47 (95% CI: 1.24–1.74) for high TC, 0.79 (95% CI: 0.64–0.96) for low HDL-C, 1.23 (95% CI: 1.03–1.45) for high LDL-C, and 1.40 (95% CI: 1.16–1.68) for high Non-HDL-C when compared with individuals in the Q1 group. In the Q4 group, multivariable-adjusted ORs were 2.06 (95% CI: 1.74–2.44) for high TC, 0.57 (95% CI: 0.46–0.70) for low HDL-C, 1.67 (95% CI: 1.41–1.98) for high LDL-C, and 1.86 (95% CI: 1.54–2.23) for high Non-HDL-C when compared with individuals in the Q1 group.

Stratified analyses were also performed using a cut-off value of 2.26 mmol/L for associations between each 1 mmol/L increase in [Ca2+]corr and dyslipidemia components. In individuals with [Ca2+]corr ≤ 2.26 mmol/L, multivariable-adjusted ORs were 11.4 (95% CI: 1.80–74.4) for high TC and 0.11 (95% CI: 0.01–0.93) for low HDL-C. In individuals with [Ca2+]corr > 2.26 mmol/L, multivariable-adjusted ORs were 37.8 (95% CI: 6.00–241) for high TC, 0.03 (95% CI: 0.00–0.39) for low HDL-C, 24.0 (95% CI: 3.78–152) for high LDL-C, and 30.40 (95% CI: 4.42–210) for high Non-HDL-C. No significant associations were identified for [Ca2+]corr with high TG (Figure 3B).

### Dose-Response Relationship Between [Ca2+]corr and Triglycerides, Total Cholesterol, High-Density Lipoprotein Cholesterol, Low-Density Lipoprotein Cholesterol, and Non-high-Density Lipoprotein Cholesterol Levels

**FIGURE 4:**
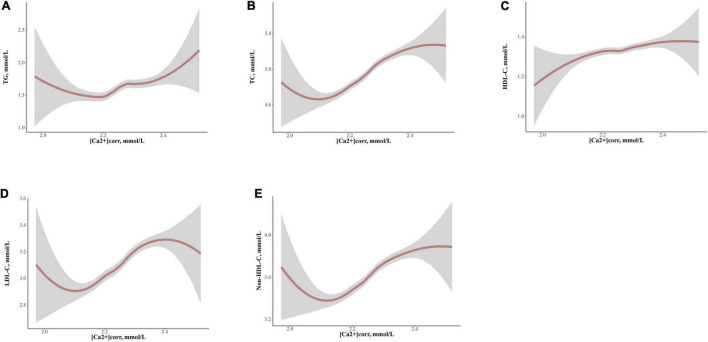
Locally weighted regression (Loess) analyses of [Ca2+]corr on serum lipid parameter levels in graphs of smoothed values with confidence bands. **(A)** TG, **(B)** TC, **(C)** HDL-C, **(D)** LDL-C, and **(E)** Non-HDL-C. [Ca2+]corr, corrected serum ionized calcium; TC, total cholesterol; TG, triglyceride; HDL-C, high-density lipoprotein cholesterol; LDL-C, low-density lipoprotein cholesterol; Non-HDL-C, non-high-density lipoprotein cholesterol.

Loess analyses were performed to explore non-linear relationships between [Ca2+]corr and serum lipid profiles (Figure 4). The five parameters, TG, TC, HDL-C, LDL-C, and Non-HDL-C had positive non-linear dependent relationships with [Ca2+]corr after adjusting for gender, age, waist circumference, BMI, SBP, DBP, a diabetes diagnosis, lymphocytes, basophils, RBC, MPV, FSG, albumin, uric acid, and ALT (all *P* < 0.05). TG displayed a W-shape relationship with [Ca2+]corr, while HDL-C showed an inverted W-shape relationship. As shown (Figures 4A,C), there was a turning point for [Ca2+]corr at 2.26 mmol/L along with TG and HDL-C, after which the dependent trend was different from before. In addition, (Figures 4B,D,E), TC, LDL-C, and Non-HDL-C showed similar sigmoidal-shaped trajectories with [Ca2+]corr, and we observed a threshold effect of [Ca2+]corr near 2.16 mmol/L for TC, LDL-C and Non-HDL-C.

## Discussion

A thorough understanding of the relationship between dyslipidemia and influencing factors is essential for effective dyslipidemia management. In this study, relationships between [Ca2+]corr and serum lipid profiles were comprehensively analyzed. We observed that [Ca2+]corr was linearly associated with higher odds of dyslipidemia, and [Ca2+]corr was positively associated with high TC, high LDL-C and high Non-HDL-C odds but inversely associated with low HDL-C odds. This is the first study to assess dose-response associations between [Ca2+]corr and dyslipidemia odds and associated components in adults, and it may be beneficial for clinical practice and public awareness of maintaining calcium nutrition at appropriate levels.

We found that [Ca2+]corr was positively linearly associated with higher odds of dyslipidemia. Similar results were reported in Italy (19) and Iran (20) on the relationship between serum calcium and dyslipidemia. However, a Chinese pediatric population study reported an L-shaped non-linear dose-response relationship between [Ca2+]corr and dyslipidemia odds (21). This difference between adults and children could be caused by differences in exogenous calcium and lipid intake and endogenous homeostatic capabilities in different age groups (22). The potential mechanism behind this positive linear association may have been because [Ca2+]corr impairs cholesterol catabolism in the liver, increases lipid synthesis (23), and impacts lipid absorption (24). This could increase the probability of high serum lipid levels and dyslipidemia *via* atherosclerotic plaque formation and an increased risk of CVD events (25). Although we used albumin corrected calcium in our study, our albumin-stratified analyses still showed that the risk of dyslipidemia attributed to [Ca2+]corr was significantly higher among people with higher albumin levels. This suggests that albumin can enhance the association between calcium and blood lipids, and that might be due to the intake of foods with high albumin levels, such as nuts, eggs, and dairy products, which are also sources of calcium and lipids (26). Moreover, our study’s [Ca2+]corr optimal cut-off level provides a quantified reference for dyslipidemia management. It could guide clinical standard practices for calcium nutrition. These findings indicated that an appropriate [Ca2+]corr level was beneficial to maintaining serum lipids at reasonable levels. Still, we also raised concerns about the adverse consequences of excessive calcium nutrition, especially for individuals taking calcium supplements (25).

In terms of dyslipidemia-associated components, high [Ca2+]corr was positively associated with increased odds of elevated TC, LDL-C, and Non-HDL-C in our study. Similar findings for the positive association between [Ca2+]corr and high TC and high LDL-C were also observed in the pediatric study (21). This finding indicated that keeping [Ca2+]corr at a reasonable level might be a new lipid treatment option, and thereby provide a potential intervention target for CVD management (27, 28). Regulation of calcium ion levels can be used as an adjunct to statin therapy for patients at high risk of CVD who need to lower LDL-C levels. Interestingly, a negative association between [Ca2+]corr and reduced HDL-C odds was also observed in our study. Although this finding agreed with Bacquer et al. (29) and Peng et al. (21), others disagreed, e.g., serum calcium was not associated with HDL-C in a study by Saltevo et al. in a general Finnish population (30) and also Chou et al. in Taiwanese community-dwelling adults (31). In our study, a non-linear association between [Ca2+]corr and HDL-C was also verified by Loess analysis. Therefore, we speculate different statistical methods in some studies may cause these inconsistencies. Specifically, Saltevo et al. (32) and Chou et al. examined associations using linear analysis methods, which may have masked non-linear differences (33). Also, while Kivela et al. reported the sterol regulatory element-binding protein (SREBP) activator, vascular endothelial growth factor A, increased HDL cholesterol levels (34), the underlying association between [Ca2+]corr and HDL-C remained unclear. These findings suggested [Ca2+]corr is a double-edged sword for serum lipid regulation. The balance of benefit and risk of [Ca2+]corr on serum lipid levels needs to be carefully weighed during clinical decision making. At the same time, the potential predictive value of [Ca2+]corr in the control of dyslipidemia, even CVD, is worth more in-depth research in the future.

As for the S-shaped relationship between [Ca2+]corr and TC LDL-C, and Non-HDL-C levels, we noted a linear increase range of the trend in 2.1–2.4 mmol/L of [Ca2+]corr and a declining trend at both ends of the drawing. A plausible explanation was the reserve capacity of the secretion of parathyroid hormone on the homeostasis of calcium regulation (35). In addition, non-linear dose-response associations between [Ca2+]corr and serum lipid profiles indicated why TG/HDL-C ratios might be more valuable than isolated lipids to predict atherogenicity (36) and CVD events (37). The underlying mechanism could be that TG/HDL-C ratios as a hinge function can magnify the scale of serum lipids change as large as possible at low and high calcium levels, therefore more sensitive to depict the covariant relations between TG and HDL-C (Supplementary Figure 1). Therefore, TG/HDL-C ratios could be used as meaningful indicators for dyslipidemia monitoring, and the clinical value of TG/HDL-C ratios in calcium or serum lipid-related diseases warrants more research.

Although the relationships between [Ca2+]corr and serum lipid profiles were comprehensively clarified, our study had limitations. Firstly, this was a cross-sectional study. Therefore, causality effects should be assessed by longitudinal studies in the future. Secondly, we included health examination individuals and not patients, so association extrapolations to patient populations should be made with caution. Thirdly, several parameters influencing [Ca2+]corr homeostasis and serum lipids levels, such as vitamin D, parathyroid hormone, and disease/medication history, were not collected. Although this study cannot rule out the influence of these factors, physical examinees, if having medication, tend to take calcium supplements and lipid-lowering medicine, which might weaken the association between [Ca2+]corr and serum lipids. We still found a significant association under conservative estimation in our study. Therefore, the impact of medication use on the association could be neglected.

In conclusion, [Ca2+]corr was positively associated with increased odds of dyslipidemia and elevated TC, LDL-C, and Non-HDL-C but inversely associated with decreased HDL-C odds among Chinese adults. Thus, [Ca2+]corr should be considered a valuable indicator for dyslipidemia management.

## Data Availability Statement

The original contributions presented in this study are included in the article/Supplementary Material, further inquiries can be directed to the corresponding authors.

## Ethics Statement

This study was approved by the Ethics Committee of the First Affiliated Hospital of China Medical University (approval No. 2020-323). Informed consent was waived as this was a retrospective study.

## Author Contributions

KY designed the study. KY, WW, and XH conducted the data collection. KY conducted the data analysis and drafted the manuscript. XH revised the manuscript. All authors read and approved the final manuscript version for publication.

## Conflict of Interest

The authors declare that the research was conducted in the absence of any commercial or financial relationships that could be construed as a potential conflict of interest.

## Publisher’s Note

All claims expressed in this article are solely those of the authors and do not necessarily represent those of their affiliated organizations, or those of the publisher, the editors and the reviewers. Any product that may be evaluated in this article, or claim that may be made by its manufacturer, is not guaranteed or endorsed by the publisher.

## Footnotes

1
http://www.r-project.org

## Supplementary Material

Supplementary Figure 1 Non-linearity relationship between [Ca2+]corr and TG/HDL-C ratio. [Ca2+]corr, corrected serum ionized calcium; TG, triglyceride; HDL-C, high-density lipoprotein cholesterol.

## Abbreviations

CVD: cardiovascular disease
TC: total cholesterol
TG: triglycerides
LDL-C: low-density lipoprotein cholesterol
HDL-C: high-density lipoprotein cholesterol
Non-HDL-C: non-high-density lipoprotein cholesterol
Ca2+: ionized calcium
[Ca2+]corr: corrected serum ionized calcium
FSG: fasting serum glucose
SBP: systolic blood pressure
DBP: diastolic blood pressure
BMI: body mass index
ALT: alanine aminotransferase
AST: aspartate aminotransferase
SCR: serum creatinine
eGFR: estimated glomerular filtration rate
RBC: red blood cells
HGB: hemoglobin concentration
PCT: plateletcrit
HCT: red blood cell specific volume
MCV: mean corpuscular volume
MCH: mean corpuscular hemoglobin
MCHC: mean corpuscular hemoglobin concentration
PLT: platelet counts
PDW: platelet distribution width
P-LCR: large platelet ratio
PCT: plateletcrit
MPV: mean platelet volume
OR: odds ratio
CI: confidence intervals
IQR: interquartile range
SD: standard deviation
AOR: adjusted odds ratio
Locally weighted regression (Loess).
