# Supplementary figures and images for: Corrected Serum Ionized Calcium as a Risk Factor Related to Adult Dyslipidemia
Source: Front Cardiovasc Med. 2022 Jul 6;9:916991. doi: 10.3389/fcvm.2022.916991 (PMC9299357; doi:10.3389/fcvm.2022.916991)

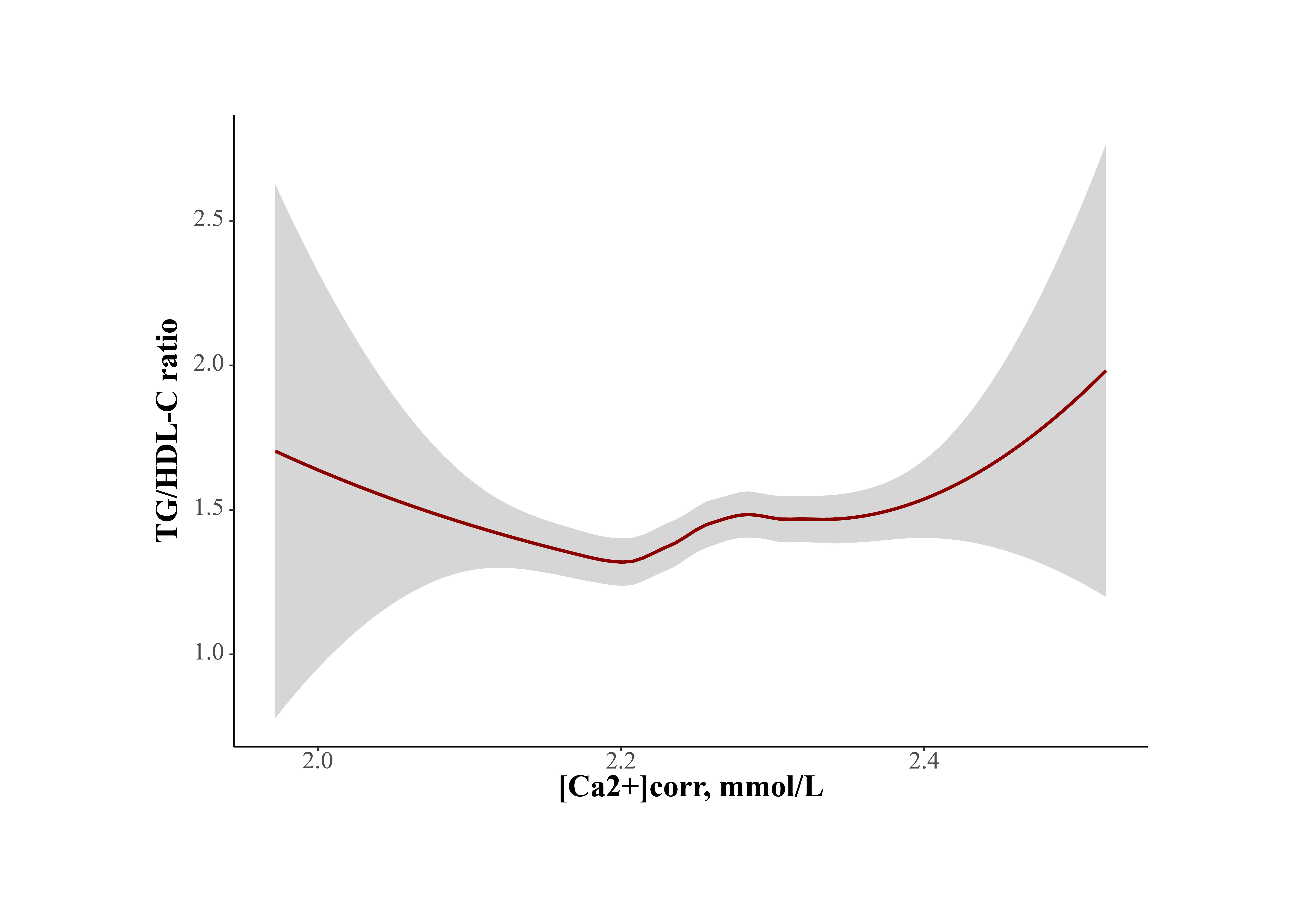

Supplement: Supplementary Figure 1 — Non-linearity relationship between [Ca2+]corr and TG/HDL-C ratio. [Ca2+]corr, corrected serum ionized calcium; TG, triglyceride; HDL-C, high-density lipoprotein cholesterol. [file Image_1.JPEG]
